# Deodorization of Spirulina Extracts by Ozone and Activated Carbon: Effects on Volatile Organic Compounds, Bioactive Pigments, Antioxidant Activity, and Sensory Profile

**DOI:** 10.3390/foods14223820

**Published:** 2025-11-07

**Authors:** Sithu Lwin, Suthat Surawang, Thanyaporn Siriwoharn

**Affiliations:** 1 Division of Food Science and Technology, Faculty of Agro-Industry, Chiang Mai University, Chiang Mai 50100, Thailand; sithulwin_s@cmu.ac.th; 2 Division of Product Development Technology, Faculty of Agro-Industry, Chiang Mai University, Chiang Mai 50100, Thailand; suthat.s@cmu.ac.th; 3 Lanna Rice Research Center, Chiang Mai University, Chiang Mai 50100, Thailand

**Keywords:** *Arthrospira platensis*, green technology, phycobiliprotein, volatile organic compounds, odor reduction, algae, functional ingredients

## Abstract

Spirulina extract holds significant promise for food applications, but its characteristic odor limits consumer acceptance. This study evaluated ozone (5, 10, and 25 ppm) and activated carbon (AC; 10, 30, and 50% *w*/*v*) treatments for their effects on selected volatile organic compounds (VOCs) in spirulina aqueous extracts, as well as on protein content, bioactive compounds, and antioxidant activities. Neither treatment adversely affected protein content. Ozone treatments significantly increased total phycobiliprotein content (172.5–181.1 mg/g; *p* < 0.05), whereas AC treatments significantly reduced it (138.5–159.0 mg/g; *p* < 0.05). Both treatments decreased chlorophyll (13.9–30.6%) and carotenoid (44.6–72.3%) levels, while DPPH and ABTS antioxidant activities varied according to treatment and concentration. AC treatments were more effective than ozone in reducing total VOCs (74.1–79.9% vs. 30.3–55.5% reduction), but 25 ppm ozone achieved the most favorable sensory profile as assessed by trained panelists. Treatments with 25 ppm ozone and 10% AC provided the best compromise between deodorization and retention of bioactive compounds. These findings indicate that both ozone and AC treatments can substantially reduce the undesirable odor of spirulina extracts, thereby improving their sensory quality and application potential in odor-sensitive food and functional products.

## 1. Introduction

Spirulina (*Arthrospira platensis*) is a blue-green microalga valued for its exceptionally high protein content (60–70 g/100 g dry weight), sustainable cultivation, and abundant bioactive compounds, especially phycocyanin, chlorophylls, and carotenoids [1,2]. These attributes position spirulina as a promising ingredient for addressing global protein needs and advancing sustainable food systems. Phycocyanin, in particular, is recognized for its vibrant color and potent antioxidant activity, supporting its use as a natural colorant and functional ingredient in food and cosmetic applications [3,4]. These qualities have driven the rapid incorporation of spirulina into various food products, such as baked goods, snacks, noodles, and confectionery, where it enhances nutritional value and imparts natural color [5,6,7,8,9,10,11,12].

However, widespread adoption of spirulina as a mainstream food ingredient remains limited by its characteristic “algal” odor, primarily arising from volatile organic compounds (VOCs) such as aldehydes, furans, and pyrazines, which negatively impact consumer acceptance [13,14,15,16]. Conventional deodorization methods—including thermal processing, solvent extraction, fermentation, and soaking—have been explored for reducing VOCs in various food matrices [17,18,19,20]. Yet, these approaches often compromise spirulina’s sensitive bioactive compounds (especially phycocyanin and chlorophylls), resulting in pigment loss, altered sensory profiles, and occasionally, residual solvents or off-flavors [13,18,19]. This challenge underscores the critical need for environmentally friendly, food-safe deodorization processes that preserve both the sensory and functional qualities of spirulina extracts.

Emerging techniques such as ozone treatment and activated carbon (AC) adsorption offer promising, green alternatives. Ozone acts as a powerful oxidizing agent that targets and degrades odorous VOCs through direct or indirect oxidation reactions, transforming them into less volatile or non-odorous compounds [21,22,23]. This gas-phase process is residue-free, minimizing the risk of chemical contaminants in the final product. In contrast, AC relies on its high surface area and porous structure to physically adsorb a broad spectrum of VOCs, effectively trapping odor-causing molecules without the use of solvents or heat, which offers a simple, cost-effective alternative in water and beverage purification [24,25,26]. Both ozone and AC treatments operate at ambient temperatures and do not require harsh chemicals, offering clear advantages over traditional approaches by reducing off-odor while better preserving pigments, proteins, and other sensitive bioactive compounds [26,27,28].

Despite their potential, no study to date has comprehensively compared ozone and AC treatments in the deodorization of spirulina aqueous extracts, particularly with respect to their effects on volatile profile, sensory attributes, and key functional properties. Therefore, the objective of this study was to systematically evaluate and compare the efficacy of ozone and AC treatments in reducing odor while preserving protein, pigment content, and antioxidant activities in spirulina extracts. These findings aim to advance the development of sustainable, consumer-accepted spirulina ingredients suitable for odor-sensitive food applications.

## 2. Materials and Methods

### 2.1. Raw Materials and Chemicals

Food-grade spirulina powder was obtained from Green Diamond Farm (Chiang Mai, Thailand). The granular AC (particle size: 1.18–2.36 mm) was purchased from Northern Chiangmai Chemical Co., Ltd. (Chiang Mai, Thailand). All chemicals and solvents used in the study were of analytical grade and acquired from Union Science Co. Ltd. (Chiang Mai, Thailand). Analytical standards, 1,1-diphenyl-2-picryl-hydrazil (DPPH), and 2,2′-azino-bis(3-ethylbenzothiazoline-6-sulfonic acid) (ABTS), were purchased from Merck KGaA (Darmstadt, Germany).

### 2.2. Extraction Procedure

A 5% (*w*/*v*) spirulina mixture was prepared by dispersing spirulina powder in distilled water. The mixture was sonicated using a high-intensity ultrasonic processor (VCX 750 W, Sonics, Newtown, CT, USA) at 20 kHz and 40% amplitude for 30 min, maintaining the temperature below 40 °C with a cooling bath to minimize degradation of bioactive compounds. Deodorization treatments (ozone or AC) were then applied as follows:

#### 2.2.1. Ozone Treatment

Ozone treatment was performed based on the method of Zhang et al. [27] with slight modifications. Ozone gas, generated using a corona discharge ozone generator (Silica tube type; Chiang Mai ETS Engineering Co., Ltd., Chiang Mai, Thailand), was pumped directly into the 200 mL aliquots of the spirulina mixture via an air diffuser (pore size: 2 μm) at a flow rate of 0.4 m^3^/h. Samples were ozonated at concentrations of 5, 10, or 25 ppm for 15 min. After ozonation, samples were rested at room temperature for 20 min to ensure complete dissipation of residual ozone, then centrifuged (6500 rpm, 30 min). The supernatant was collected, adjusted to a known volume with distilled water, and stored at −20 °C until analysis.

#### 2.2.2. Activated Carbon (AC) Treatment

Granular AC was added to the spirulina mixture at AC: mixture ratios of 1:10, 3:10, or 5:10 (*w*/*v*) based on the method of Khalafu et al. [26] with slight modification. Each sample was agitated on an orbital shaker (ProfiLab24 GmbH, Berlin, Germany) for 30 min at room temperature. The AC was removed by filtration through nylon filter cloth (75 µm; Rever Store, Bangkok, Thailand), followed by centrifugation (6500 rpm, 30 min). The supernatant was collected, adjusted to a known volume with distilled water, and stored at −20 °C until analysis.

### 2.3. Determination of Protein Content

Untreated (control) and treated extracts were lyophilized using a FreeZone 4.5 L freeze dryer (Labconco, Kansas City, MO, USA). Protein content (% *w*/*w*, dry weight basis) in the spirulina powder and lyophilized extracts was determined using the Dumas combustion method on a TruMac N protein analyzer (Leco Corp., St. Joseph, MI, USA), following the AOAC method 990.03 [29]. Nitrogen content was measured, and protein content was calculated as:

$$Protein\left(\%\right)=Nitrogen\left(\%\right)\times 6.25$$

### 2.4. Determination of Total Phycobiliprotein Content

Total phycobiliprotein content was determined according to the method of Pan-utai and Iamtham [30], with minor modifications. Briefly, 1 mL of extract was diluted 100-fold with distilled water. Aliquots were transferred to a 96-well plate, and absorbance (OD) was measured at 562, 620, and 652 nm using a microplate reader (Spark^®^ 10M multimode, Tecan Trading AG, Männedorf, Switzerland). The contents of phycocyanin (PC), allophycocyanin (APC), and phycoerythrin (PE) were calculated according to the following equations and expressed as mg per g of spirulina powder:

$$PC=\frac{{OD}_{620}-0.474\left({OD}_{652}\right)}{5.34}$$

$$APC=\frac{{OD}_{652}-0.208({OD}_{620})}{5.09}$$

$$PE=\frac{{OD}_{562}-2.41(PC)-0.849(APC)}{9.62}$$

Total Phycobiliprotein=PC+APC+PE

### 2.5. Determination of Total Chlorophyll and Total Carotenoid Contents

Total chlorophyll and total carotenoid contents were determined spectrophotometrically following the method of Lichtenthaler and Buschmann [31]. Briefly, 1 mL of extract was mixed with 4 mL of methanol, vortexed at room temperature, and centrifuged at 5000 rpm for 10 min. The resulting supernatant was collected, and absorbance was measured at 470, 652, and 665 nm using a microplate reader, with methanol as a blank. The contents of chlorophyll a (Chl a), chlorophyll b (Chl b), total chlorophyll, and total carotenoid were calculated using the following equations and expressed as mg per g of spirulina powder:

$$Chl\;a=16.72\;{OD}_{665}-9.28\;{OD}_{652}$$

$$Chl\;b=36.92\;{OD}_{652}-9.28\;{OD}_{665}$$

Total Chlorophyll=Chl a+Chl b

$$Total\;Carotenoid=\frac{\left(1000\;{OD}_{470}-1.91\;Chl\;a-95.15\;Chl\;b\right)}{225}$$

### 2.6. Determination of Antioxidant Activities

#### 2.6.1. DPPH Assay

The DPPH assay was performed using the method described by Brand-Williams et al. [32], with minor modifications. Briefly, a 0.1 mM DPPH solution was prepared in methanol. For each analysis, 25 μL of extract was mixed with 200 μL of DPPH solution in a 96-well microplate. The mixture was shaken, incubated at room temperature for 30 min, and absorbance was measured at 517 nm using a microplate reader. Methanol was used as a blank. DPPH antioxidant activity (DPPH activity) was calculated based on a Trolox standard curve and expressed as mg Trolox equivalents per g of spirulina powder (mg TE/g).

#### 2.6.2. ABTS Assay

The ABTS assay was conducted according to the method of Chen et al. [33], with minor modifications. ABTS (7 mM) was dissolved in distilled water and reacted with 2.45 mM potassium persulfate in the dark at room temperature for 16 h to generate ABTS radical cations. The resulting solution was diluted with distilled water to an absorbance of 0.70 ± 0.02 at 734 nm and equilibrated at room temperature. For analysis, 25 μL of extract was mixed with 175 μL ABTS solution in a 96-well microplate and incubated at room temperature for 7 min. Absorbance at 734 nm was measured using a microplate reader. Methanol was used as the blank. ABTS antioxidant activity (ABTS activity) was calculated using a Trolox standard curve and reported as mg TE/g.

### 2.7. Determination of Volatile Organic Compounds (VOCs)

The VOCs in spirulina samples were analyzed by headspace gas chromatography–tandem mass spectrometry (HS-GC–MS/MS). Analyses were performed on an EVOQ GC-TQ MS system (Bruker, Billerica, MA, USA) equipped with a multi-purpose autosampler (GERSTEL GmbH & Co. KG, Mülheim an der Ruhr, Germany). For each analysis, 5 mL of extract was transferred into a 20 mL screw-cap glass vial with PTFE/silicone septa and incubated at 70 °C for 15 min. A 2.5 mL sample of headspace gas was withdrawn automatically using a 65 mm gas-tight syringe and injected into the GC in split mode (split ratio: 10:1).

Chromatographic separation was performed on a Rxi-5Sil MS quartz capillary column (30 m × 0.25 mm i.d., 0.25 μm film thickness; Restek Corp., Bellefonte, PA, USA) with helium as the carrier gas at a flow rate of 1 mL/min. The oven temperature program was as follows: 45 °C for 2.5 min; ramped to 80 °C at 10 °C/min (held for 3 min); ramped to 200 °C at 15 °C/min (held for 1 min); and finally ramped to 250 °C at 20 °C/min (held for 30 s). The mass spectrometer was operated in electron ionization (EI) mode at 70 eV with a scan range of *m*/*z* 45–350.

VOCs were identified by comparison to the National Institute of Standards and Technology (NIST) 2020 mass spectral library, using an 80% minimum similarity threshold. The relative content of each compound was calculated by integrating the respective peak areas and expressing it as a percentage of the total chromatographic peak area. Headspace vials without a sample (blanks) were run as negative controls.

### 2.8. Orthonasal Sensory Evaluation

The orthonasal sensory evaluation of spirulina was conducted as described by Meilgaard et al. [34]. Eight trained panelists were screened and then trained using developed reference standards for five odor descriptors: fishy, green/leafy, seaweed/ algae, fatty/rancid, and charcoal/smoke. Panelists assessed the sensory qualities of both untreated (control) and treated spirulina samples. Each sample (1 g extract in 10 mL water) was presented at room temperature in lidded amber headspace containers, labeled with unique three-digit random codes, and served in a randomized order. During the descriptive sensory analysis, panelists matched odor profiles for qualitative attributes and rated intensity on a 5-cm continuous scale (0 = none, 5 = extremely strong). A 1-min resting interval was maintained between samples, during which panelists sniffed coffee beans as a palate cleanser. Results were expressed as quantitative values and displayed using spider network diagrams. The sensory evaluation protocol was approved by the Chiang Mai University Research Ethics Committee (CMUREC; No. 67/216).

### 2.9. Statistical Analysis

All measurements were performed in triplicate, and results are expressed as mean ± standard deviation. Statistical significance was determined using one-way analysis of variance (ANOVA) followed by Duncan’s Multiple range test, with differences among means considered significant at *p* < 0.05. All statistical analyses were conducted using SPSS version 17.0 software (SPSS Inc., Chicago, IL, USA).

## 3. Results and Discussion

### 3.1. Protein Content

**Figure 1:**
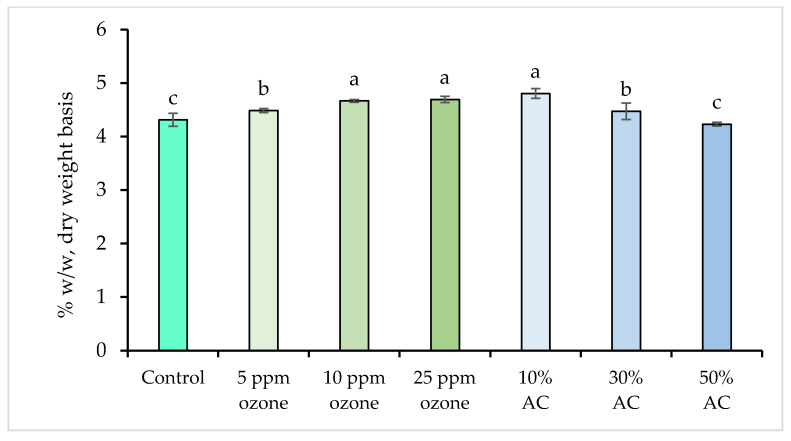
Protein content (% *w*/*w*, dry weight basis) in spirulina extracts after ozone and activated carbon (AC) treatments. Different letters indicate significant differences among treatments (*p* < 0.05).

The initial spirulina raw material contained 68.38 ± 1.09% (*w*/*w*) protein, consistent with previous reports [2,35,36]. Extraction yielded a control extract containing 4.31 ± 0.12% protein, equivalent to 6.30% recovery of the initial protein content. Application of ozone or AC during extraction produced a statistically significant increase in protein content compared to the control (*p* < 0.05; Figure 1), with maximum values of 4.89 ± 0.04% for 10% AC, 4.67 ± 0.03% for 10 ppm ozone, and 4.69 ± 0.06% for 25 ppm ozone. These treatments, however, were not significantly different from each other (*p* > 0.05).

Although statistically significant, the absolute protein increase was minimal, with a maximum difference of ~0.6% compared to the control. This suggests that ultrasonic extraction was the primary factor for protein recovery under these conditions, while ozone and AC contributed only marginally. The slight increases may be due to minor cell wall disruption by ozone or adsorption/desorption dynamics related to AC, but these effects did not translate to notable practical improvements. Importantly, while their contribution to extraction efficiency was negligible, ozone and AC treatments did not negatively impact protein yields, indicating that both can be incorporated into spirulina extraction protocols without concern for protein loss.

Notably, the majority of proteins remained in the biomass residue after extraction. This highlights a significant opportunity for process improvement to enhance protein yield. Future research should focus on optimizing extraction conditions and exploring sustainable strategies, such as enzyme-assisted extraction [37] or pulsed electric field-assisted extraction [38], both of which have been shown to substantially improve protein recovery from microalgae, thereby increasing the value and sustainability of spirulina-based food and nutraceutical products.

### 3.2. Bioactive Pigments and Antioxidant Activities

#### 3.2.1. Phycobiliprotein Content

**Figure 2:**
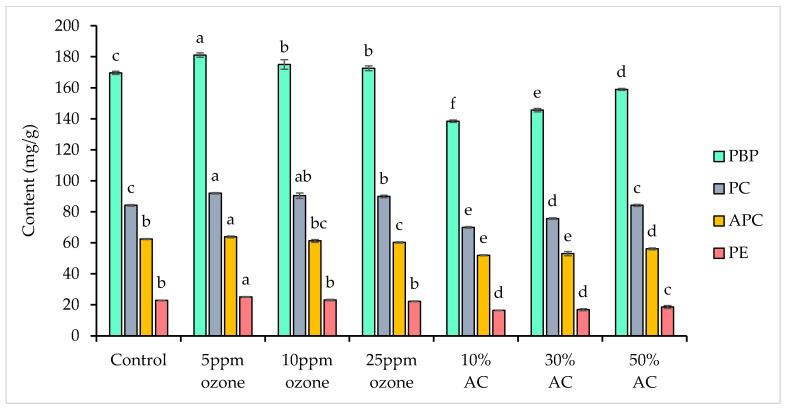
Phycobiliprotein content (mg/g) in spirulina extracts after ozone and activated carbon (AC) treatments. Different letters indicate a significant difference among treatments for the same compound (*p* < 0.05). PBP = total phycobiliprotein, PC = phycocyanin, APC = allophycocyanin, PE = phycoerythrin.

Phycobiliproteins (PBPs) are water-soluble, light-harvesting protein-pigment complexes that are covalently attached to the thylakoid membranes of spirulina, where they play key roles in photosynthesis [30]. In this study, the control contained a total phycobiliprotein content of 169.61 ± 1.00 mg/g, consisting of 84.26 ± 0.53 mg/g PC, 62.46 ± 0.31 mg/g APC, and 22.89 ± 0.19 mg/g PE. In all extracts, PC was the most abundant phycobiliprotein, followed by APC and then PE (Figure 2).

Ozone treatments significantly increased total phycobiliprotein content (172.54–181.10 mg/g; *p* < 0.05) compared to both the control and AC-treated extracts, with the highest yield observed in the 5 ppm ozone treatment (181.10 ± 1.37 mg/g). This enhancement is likely attributable to the disruption of the spirulina cell wall, which consists predominantly of glucan and peptidoglycan polymers [39]. It has been proposed that ozone and its reactive oxygen species may interact with these cell wall components, increasing permeability and facilitating the release of intracellular compounds such as phycobiliproteins [21,28,40,41]. While the precise mechanism of cell wall rupture by ozone in spirulina is not fully established, this hypothesis is supported by studies on other microalgae and microbial inactivation, which have reported oxidative modification of cellular structures under ozone treatment [21,28,40]. However, at higher ozone concentrations, the content of extractable phycobiliproteins declined, presumably due to oxidative degradation and breakdown of the released proteins. This concentration-dependent effect aligns with literature findings that optimal ozone dosages maximize extraction efficiency while minimizing oxidative loss of bioactive compounds [41,42].

Conversely, all AC-treated extracts had significantly lower total phycobiliprotein content than both the control and ozone-treated groups (*p* < 0.05), although values increased as the AC concentration was raised from 10% to 50%. Losses were greatest for PE (18.5–27.9%), followed by APC (10.1–16.8%), and lowest for PC (0.0–16.9%). The observed pattern—greatest losses for PE, intermediate for APC, and lowest for PC—may reflect both selective adsorption by AC and differences in the molecular sizes of the phycobiliproteins [43]. PE, being the largest complex (240–290 kDa), followed by APC (105 kDa) and PC (70–110 kDa) [44], is therefore more susceptible to removal by AC.

The surface of AC contains various oxygen-containing functional groups (e.g., carboxyls, lactones, phenols), which influence its surface acidity and charge [25]. Under acidic conditions, these groups may impart a positive or less negative charge to the carbon, promoting electrostatic attraction with anionic molecules and selective adsorption of proteins, dependent on their isoelectric point and the pH of the medium [25,43,45]. These charge interactions and surface properties likely play a central role in the observed losses of phycobiliproteins. This corresponds with the observed changes in extract color (Table S1).

This trend contrasts with the findings of Payne [43], who reported a reduction in phycocyanin recovery as AC powder concentration increased from 6% to 10%. The discrepancy may result from differences in AC form (powder vs. granular) and/or other methodological variables, emphasizing that the impact of AC on phycobiliprotein recovery can be both form- and concentration-dependent. Overall, ozone treatment is more effective than AC, where retention of phycobiliproteins in spirulina extracts is desired. These findings highlight the importance of selecting deodorizing strategies tailored to applications in which phycobiliprotein retention is essential, such as natural colorants and functional foods. The pronounced loss of phycobiliproteins during AC treatment underscores the need to carefully optimize AC concentration to minimize such losses and preserve the functional quality of spirulina extracts.

#### 3.2.2. Total Chlorophyll and Total Carotenoid Contents

Chlorophylls and carotenoids are important lipid-soluble bioactive compounds in spirulina. Although they are not volatile compounds, their degradation—especially by oxidative or enzymatic processes—generates a range of VOCs that contribute to the odor profile of spirulina extracts [14,15,46]. Despite their lipophilicity, small amounts were detected in the aqueous extracts (Table 1), likely due to partial solubilization and/or association with soluble proteins or membranes. Both ozone and AC treatments significantly reduced the content of these compounds compared to the control (*p* < 0.05), except that 10% AC preserved total chlorophyll content and 25 ppm ozone preserved total carotenoid content at levels not significantly different from the control (*p* > 0.05).

AC treatments were more effective at preserving total chlorophyll content (80.6–100.0% remaining) than ozone treatments (69.4–75.0% remaining). Chlorophylls appeared to be more sensitive to oxidative degradation by ozone than to physical adsorption by AC. The observed decrease in total chlorophyll content after ozone treatment is attributable to ozone’s strong oxidative properties. In aqueous solution, ozone rapidly decomposes into reactive oxygen species such as hydroxyl (HO•) and hydroperoxyl (HO_2_•) radicals, which accelerate the degradation of chlorophyll molecules [21,47]. In contrast, the AC mechanism operates primarily via weak interactions such as van der Waals forces, hydrogen bonding, and π–π stacking [25,48,49]. Additionally, the reduction in chlorophylls may partially result from enzymatic reactions involving the action of chlorophyllase, Mg-dechelatase, and pheophorbide oxygenase, which subsequently degrade chlorophyll molecules [47,50]. Rame et al. [51] also observed reduced chlorophyll yields in *Chlorella vulgaris* pretreated with increased ozone concentrations, while Han et al. [52] reported similar ozone-dependent chlorophyll losses in Brassica leaves, although carotenoid content remained unaffected.

Similar to total chlorophyll content, the reduction in total carotenoid content may be attributed to the oxidative effects of ozone and to competition and selectivity among co-extracted compounds during AC adsorption [47,49,53]. Overall, the total carotenoid content was higher in the ozone-treated extracts (68.6–102.8% remaining) than in the AC-treated extracts (51.4–82.8% remaining), with higher treatment concentrations enhancing carotenoid retention in both groups. These findings are important for optimizing spirulina extract processing to retain the bioactive profile best suited to target food applications. Developing strategies to minimize the degradation of chlorophylls and carotenoids could also reduce the formation of unwanted VOCs, thereby improving the odor and quality of spirulina extracts.

#### 3.2.3. Antioxidant Activities

The antioxidant activities of extracts typically reflect the combined effects of their bioactive compounds. In this study, ozone treatments and 10% AC treatment did not significantly affect DPPH activity compared to the control (*p* > 0.05; Table 1), whereas higher AC concentrations led to a decrease in DPPH activity. In contrast, both ozone and AC treatments significantly influenced ABTS activity in a concentration-dependent manner (*p* < 0.05; Table 1). Notably, ABTS activity was 4.3- to 6.5-fold higher than DPPH activity, consistent with the greater sensitivity of ABTS radical cation to hydrophilic and lipophilic antioxidants, whereas DPPH radical is primarily sensitive to hydrophobic compounds and less reactive toward hydrophilic antioxidants [32,54,55,56]. This difference is attributable to the distinct chemical nature of the radicals involved. The DPPH assay is based on electron/hydrogen-donating activity in a relatively hydrophobic environment, while the ABTS assay allows more access to hydrophilic and larger molecular antioxidants. Phycobiliproteins, which are major pigments in these extracts, have demonstrated both DPPH and ABTS activities, with stronger correlations observed for ABTS activity in aqueous contexts [57,58].

Among all treatments, 5 ppm ozone provided the best overall antioxidant activities, as measured by both DPPH (3.44 ± 0.23 mg TE/g) and ABTS (19.01 ± 0.55 mg TE/g) assays, compared to the control and AC treatments. However, higher ozone concentrations reduced antioxidant activities, likely due to degradation of bioactive compounds. This trend was also observed by Matłok et al. [59] in alligator plant extracts, and similar reductions were reported in fruit juices after prolonged ozone treatment [60,61]. This reduction in ABTS activity at higher ozone doses suggests a strong correlation with phycobiliprotein content, as these water-soluble pigments contribute predominantly to ABTS rather than DPPH activity.

The effects of AC were assay-dependent: increasing AC concentration decreased DPPH activity but increased ABTS activity (Table 1). Such differences may relate to the type and quantity of retained bioactive compounds, the selective adsorption characteristics of AC, and the differing reactivity and solubility of compounds in the two assay systems [25,43]. The decrease in DPPH activity is likely due to the reduced hydrogen-donating capacity of retained compounds following AC adsorption [26,43,62]. Similar declines in antioxidant activity after AC treatment have been reported, including a 30–65% decrease in cashew leaf extracts [62], a 90% decrease in peanut protein hydrolysates at higher pH [63], and notable reductions in apple cider [64], attributed to selective adsorption of bioactive compounds. However, the increase in ABTS activity may result from the additive effects of phenolic acids and flavonoids present in spirulina [65]. Further analysis of total phenolic and flavonoid content, as well as HPLC profiling, would be valuable for elucidating the contributions of these compounds to the overall antioxidant activity of spirulina extracts.

### 3.3. Volatile Organic Compounds (VOCs)

**Figure 3:**
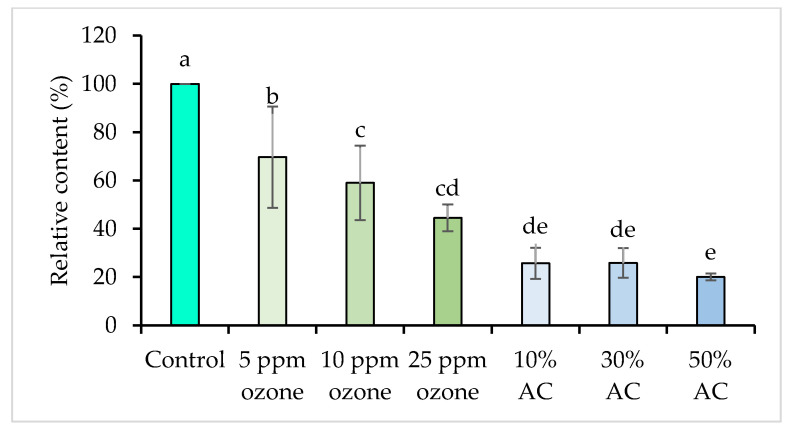
Total volatile organic compound content in spirulina extracts after ozone and activated carbon (AC) treatments. Different letters indicate significant differences among treatments (*p* < 0.05).

VOCs have been associated with spirulina’s characteristic, unpleasant, algal odor. Both ozone and AC treatments significantly reduced the total VOCs content in the spirulina extracts compared to the control (*p* < 0.05; Figure 3). Total VOCs content decreased with increasing ozone concentration, with 25 ppm ozone achieving the greatest reduction among ozone treatments (55.5%) relative to the control. In contrast, all concentrations of AC resulted in a greater reduction in total VOCs content (74.11–79.95%) than ozone treatments. These results indicate that AC adsorption is more effective than ozone (at 5–25 ppm) in reducing total VOCs content in spirulina extracts.

These differences may reflect both the distinct mechanisms of VOCs removal and the stability of ozone in aqueous solution. AC is considered a non-selective adsorbent because it interacts with a broad range of molecules through generalized surface processes. VOCs adsorption occurs via hydrophobic interactions, van der Waals forces, hydrogen bonds, and π–π stacking, particularly involving the polyaromatic sheet structure of carbon [25,45,48,49]. By contrast, ozone removes VOCs primarily through direct chemical oxidation, such as ozonolysis of double bonds in volatile compounds [21,22,28]. Moreover, ozone is partially soluble in water [21], and the concentration of active ozone in aqueous extracts decreases gradually over time due to its chemical instability and rapid decomposition [66]. In comparison, AC remains fully immersed throughout the treatment, continuously and efficiently adsorbing target compounds from the solution. Previous studies confirm that ozone concentrations in water diminish due to decomposition during extended treatment durations [66].

VOCs analysis of the control extract identified 27 compounds (Table 2), consisting of six aldehydes, four ketones, four alcohols, two esters, four heterocyclic compounds, five hydrocarbons, and two additional compounds (hexathiane [sulfur S6] and dodecamethyl-cyclohexasiloxane). Among these, 3-methyl-butanal, hexanal, 1-octen-3-ol, 6-methyl-5-hepten-2-one, and 2-pentyl-furan are established contributors to undesirable spirulina odor [14,22,23,67]. Therefore, these compounds were selected as key targets for evaluating deodorization treatments.

3-Methyl-butanal is associated with malty, fermented odors and likely originates from amino acid metabolism in spirulina [22]. Its higher content (Table 3) may be attributed to protein degradation via Maillard reactions, potentially promoted by mechanical effects during ultrasonic extraction [67,68]. Hexanal, responsible for grassy and green odors, is typically generated by fatty acid oxidation [15]. 1-Octen-3-ol, a key fishy, mushroom-like odorant in algae and cyanobacteria, may result from lipid oxidation and chlorophyll degradation following cell breakage during ultrasonication [15,68]. 6-Methyl-5-hepten-2-one is formed by the oxidative cleavage of carotenoids, particularly lycopene and phytoene [14,68]. 2-Pentyl-furan is a frequent contributor to the sensory profile of spirulina [15].

Ozone treatments significantly decreased the concentrations of hexanal (by 49.9–70.3%) and 2-pentyl-furan (by 75.6–86.6%) and were more effective than the control and AC treatments (*p* < 0.05; Table 3). This effect is likely due to the high reactivity of ozone with double bonds and furan derivatives, resulting in their breakdown to smaller or less volatile compounds [21,22,23]. However, more saturated or sterically hindered odorants, such as 3-methyl-butanal and 1-octen-3-ol, are less reactive toward ozone, which may account for the insignificant reduction (*p* > 0.05) in these cases. Similar reductions in hexanal and 1-octen-3-ol following ozone treatment have been reported in Nile tilapia mince [23] and grass carp surimi [22], respectively.

With AC treatments, significant reductions were observed for 3-methyl-butanal, hexanal, 1-octen-3-ol, and 6-methyl-5-hepten-2-one compared to the control (*p* < 0.05), but not for 2-pentyl-furan. For hexanal, 10% AC was more effective than 30% or 50% AC. Notably, an increase in hexanal at higher AC concentrations may be attributed to unfavorable pH-induced changes in AC surface chemistry [25,69]. Additionally, the release of trapped air bubbles from the AC upon immersion, combined with reduced phycobiliprotein content in the extract, may catalyze lipid oxidation, leading to increased hexanal formation [46,62]. For other target VOCs, no significant differences among AC concentrations were detected (*p* > 0.05), suggesting that lower AC concentrations are generally sufficient.

In the present study, AC treatments were 4.3–42.2 times more effective than ozone in removing 3-methyl-butanal, reflecting the contrasting mechanisms of ozone oxidation and AC adsorption. AC predominantly removes VOCs by adsorption, a process influenced by the surface area, pore size distribution, and surface chemistry of the carbon, as well as by the physicochemical properties of the VOCs [25]. In this study, AC selectively and effectively removed aldehydes and small alcohols, as indicated by the marked reductions in 3-methyl-butanal and 1-octen-3-ol. Chen et al. [70] also reported significant reductions in hexanal and other aldehydes and ketones in *Paphia undulata* hydrolysate treated with AC, although 1-octen-3-ol was not significantly affected. Overall, the reduction in target VOCs by AC and ozone treatments is expected to enhance the odor profile of spirulina extracts by minimizing undesirable notes.

### 3.4. Sensory Evaluation

**Figure 4:**
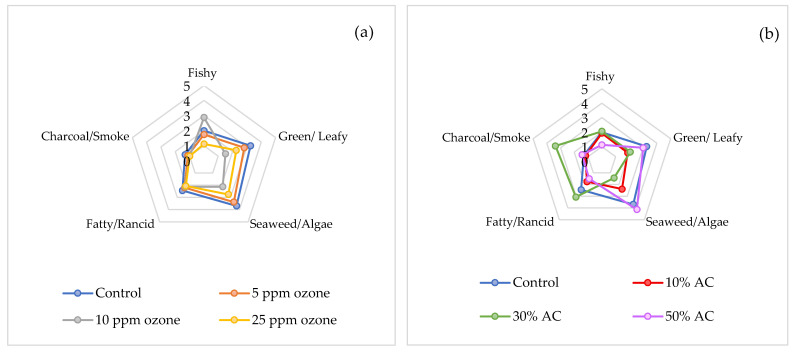
Spider diagram of sensory evaluation in odor intensity of untreated (control) and treated spirulina extracts; (**a**) after ozone treatment for 15 min; (**b**) after activated carbon treatment (AC) for 30 min; average scores are shown after quantitative descriptive sensory evaluation by 8 trained panelists.

Eight trained panelists evaluated the “fishy,” “green/leafy,” “seaweed/algae,” “fatty/rancid,” and “charcoal/smoke” odor attributes of the spirulina extracts. Table 4 and Figure 4 present the average sensory scores for untreated (control) and treated samples. Both ozone and AC treatments significantly reduced the perceived intensity of all target odor attributes in spirulina extracts compared to the control (*p* < 0.05), except for the fatty/rancid and charcoal/smoke attributes. The fatty/rancid attribute was significantly reduced only by the 50% AC treatment (38.5% decrease; *p* < 0.05). The charcoal/smoke attribute did not show a significant reduction for any treatment (*p* > 0.05); moreover, the 30% AC treatment produced a significantly higher charcoal/smoke intensity than the control (*p* < 0.05), indicating a potential undesirable effect for this attribute. The charcoal/smoke odor, associated with a burnt-nutty smell, may originate from pyrazine compounds formed via the Maillard reaction [68] or from AC itself.

The fishy odor, associated with 3-methyl-butanal and 1-octen-3-ol [68], was scored as the least intense in spirulina extracts treated with the highest concentrations of ozone (25 ppm) and AC (50%). In contrast, the green/leafy odor, attributed to hexanal and 2-pentyl-furan [14], showed the greatest reduction in intensity with the 10 ppm ozone (53.8% decrease) and 10% AC (42.2% decrease) concentrations. For the seaweed/algae attribute, the 30% AC treatment was the most effective in reducing this odor, followed by 10 ppm ozone, 10% AC, and 25 ppm ozone. In this study, panelists perceived significantly lower overall odor intensity relative to the control in extracts treated with all ozone concentrations and the 10% AC treatment (*p* < 0.05).

Although AC treatment was more effective than ozone at reducing total VOCs, sensory evaluation did not show a directly corresponding improvement. This discrepancy likely arises because each sensory attribute results from the combined effects of numerous volatile compounds, rather than from a limited set of target VOCs. Furthermore, it is well established that relating human flavor perception to analytical profiles of volatiles is inherently challenging, as the interactions and synergistic effects among different compounds can yield sensory impressions distinct from individual component contributions [71]. Nonetheless, both ozone and AC treatments effectively reduced undesirable odors in spirulina extracts, particularly diminishing fishy, green/leafy, and seaweed/algae attributes. Future research should employ multivariate techniques such as Principal Component Analysis (PCA) or Partial Least Squares (PLS) regression to more rigorously explore and quantify correlations between VOCs and sensory attributes.

Ozone treatment is approved by the U.S. Food and Drug Administration for use as an antimicrobial agent in food processing (21 CFR 173.368; [72]) and is recognized by the European Food Safety Authority (EFSA) [73] as a disinfectant and antimicrobial agent under regulated conditions. In this study, we applied aqueous ozone concentrations ranging from 5 to 25 ppm, which are lower than those previously used for antimicrobial applications in orange juice [74] and beef [72] and for odor reduction in Nile tilapia mince [23], to minimize potential impacts on bioactive compounds. Ozone rapidly decomposes to oxygen, leaving no harmful chemical residues on food products [21,73]. Regulatory assessments and prior studies indicate that, when applied under good manufacturing practices, ozone does not pose a risk to consumer health [72]. However, precautions are necessary during ozone operation, as the Occupational Safety and Health Administration (OSHA) sets the permissible exposure limit at 0.1 ppm averaged over an 8-h work shift [21,72]. Consequently, rigorous controls—including containment, ventilation, real-time ozone monitoring, and thorough post-treatment degassing—are essential to safeguard operator health and ensure compliance with workplace safety regulations.

## 4. Conclusions

This study demonstrated that both ozone and AC treatments improve the quality of spirulina aqueous extracts by effectively reducing undesirable VOCs while preserving protein content and key bioactive pigments. Ozone treatment uniquely enhanced total phycobiliprotein content, whereas AC achieved the greatest overall reduction in VOCs. Although both treatments influenced chlorophyll, carotenoid, and antioxidant activities, 25 ppm ozone and 10% AC offered the optimal balance between deodorization and nutritional preservation. Among these, 25 ppm ozone provided the most favorable sensory profile. Customizing deodorizing process conditions to balance nutritional value and sensory properties will be essential to broaden the utilization of spirulina extracts in the food industry. Future studies should investigate the incorporation of these treated spirulina extracts into various food products, evaluating their functionality, sensory acceptance, and impact on product quality.

## Figures and Tables

**Table 1:** Total chlorophyll content, total carotenoid content, and antioxidant activities in spirulina extracts after ozone and activated carbon (AC) treatments.

Sample	Total Chlorophyll (mg/g)	Total Carotenoid (mg/g)	DPPH Activity (mg TE/g)	ABTS Activity (mg TE/g)
Control	0.36 ^a^ ± 0.01	0.35 ^a^ ±0.02	3.37 ^a^ ± 0.08	14.41 ^d^ ± 0.23
5 ppm ozone	0.26 ^e^ ± 0.01	0.24 ^c^ ± 0.01	3.44 ^a^ ± 0.23	19.01 ^a^ ± 0.55
10 ppm ozone	0.27 ^d^ ± 0.01	0.31 ^b^ ± 0.01	3.39 ^a^ ± 0.19	14.47 ^d^ ± 0.55
25 ppm ozone	0.25 ^e^ ± 0.00	0.36 ^a^ ± 0.02	3.28 ^a^ ± 0.12	14.81 ^cd^ ± 0.35
10% AC	0.36 ^a^ ± 0.01	0.18 ^d^ ± 0.02	3.45 ^a^ ± 0.24	14.86 ^cd^ ± 0.12
30% AC	0.31 ^b^ ± 0.00	0.26 ^c^ ± 0.00	2.63 ^b^ ± 0.21	17.20 ^b^ ± 0.12
50% AC	0.29 ^c^ ± 0.00	0.29 ^b^ ± 0.00	2.51 ^b^ ± 0.21	15.40 ^c^ ± 0.78

Different letters indicate a significant difference (*p* < *0.05*) within the same column. TE = Trolox equivalent.

**Table 2:** Identified volatile organic compounds in spirulina extract.

No.	Retention Time (min)	Compounds	Odor Description *	Similarity Index (%)
1	1.35	2,3-butanedione	Buttery	96.7
2	1.71	3-methyl-butanal	Fishy, malty, fermented	95.7
3	2.01	4-octenoic acid, ethyl ester (Z)	n/a	88.3
4	2.15	3-hydroxy-2-butanone	Buttery	92.8
5	2.56	Isoamyl alcohol	Acetone-like, fruity, banana-like	83.9
6	3.49	Hexanal	Fatty green, grassy	88.9
7	4.55	1,2,4,4-tetramethylcyclopentene	n/a	66.8
8	4.72	1-hexanol	Grassy	82.3
9	5.35	Heptanal	Citrus-fruit	91.0
10	6.48	Benzaldehyde	Bitter nutty	85.0
11	6.95	1-octen-3-ol	Mushroom-like, earthy, soapy	91.7
12	7.03	6-methyl-5-hepten-2-one	Mushroom-like, pepper	95.8
13	7.14	2-pentyl-furan	Fruity, beany, green, metallic, vegetable, earthy	93.0
14	7.56	Trimethyl pyrazine	Earthy, nutty	96.6
15	8.39	2,2,6-trimethyl-cyclohexanone	Camphor-like, fruity, floral	95.9
16	9.21	Isophorone	Woody	82.1
17	9.96	Tetramethyl pyrazine	Roasted, nutty, fermented	98.5
18	10.34	2-ethylidene-6-methyl-3,5-heptadienal	Fruity, green, fatty	81.1
19	11.41	4-ethyl-2,5,6 trimethyl pyrimidine	n/a	80.0
20	12.09	Dodecane	n/a	79.5
21	12.36	β-cyclocitral	Sweet-tobacco, fruity	88.0
22	13.41	Dodecamethyl-cyclohexasiloxane	n/a	92.9
23	14.88	Tricyclo[4.2.2.0(2,5)]deca-7,9-diene-7,8-dicarboxylic acid, 3-cyano-, dimethyl ester	n/a	84.6
24	15.34	Pentadecane	Waxy, oily, petroleum-like	89.0
25	15.54	Hexathiane (Sulfur S6)	Sulfur-like, pungent	80.0
26	16.18	Hexadecane	Mild, waxy	91.4
27	16.96	Nonadecane	Petroleum-like	92.6

* Based on previous studies [14,15]; n/a = no description available.

**Table 3:** Relative content (%) of selected volatile organic compounds in spirulina extracts after ozone and activated carbon (AC) treatments.

Treatment	3-methyl-butanal	hexanal	1-octen-3-ol	6-methyl-5-hepten-2-one	2-pentyl-furan
Control	29.57 ^a^ ± 6.69	8.22 ^b^ ± 1.29	2.52 ^a^ ± 1.48	1.55 ^a^ ± 1.32	1.72 ^a^ ± 1.15
5 ppm ozone	25.51 ^a^ ± 3.55	4.12 ^d^ ± 0.76	1.48 ^ab^ ± 0.30	0.73 ^ab^ ± 0.15	0.42 ^b^ ± 0.16
10 ppm ozone	29.05 ^a^ ± 1.71	3.89 ^d^ ± 0.36	1.49 ^ab^ ± 0.12	0.70 ^ab^ ± 0.25	0.24 ^b^ ± 0.25
25 ppm ozone	28.21 ^a^ ± 1.73	2.44 ^e^ ± 0.68	1.42 ^ab^ ± 0.06	0.75 ^ab^ ± 0.30	0.23 ^b^ ± 0.21
10% AC	11.93 ^b^ ± 0.53	6.68 ^c^ ± 0.20	0.71 ^b^± 0.19	0.41 ^b^ ± 0.06	1.37 ^a^ ± 0.15
30% AC	9.81 ^b^ ± 1.14	10.58 ^a^ ± 0.35	1.57 ^ab^ ± 0.92	0.72 ^ab^ ± 0.31	1.48 ^a^ ± 0.33
50% AC	7.65 ^b^ ± 5.06	9.74 ^a^ ± 0.31	0.89 ^b^ ± 0.22	0.62 ^ab^ ± 0.13	1.66 ^a^ ± 0.21

Different letters indicate a significant difference (*p* < 0.05) within the same column.

**Table 4:** Odor intensity scores of spirulina extracts after ozone treatment for 15 min and activated carbon treatment (AC) for 30 min.

Treatments	Fishy	Green/Leafy	Seaweed/Algae	Fatty/Rancid	Charcoal/Smoke
Control	2.00 ^b^ ± 0.53	3.25 ^a^ ± 0.46	3.69 ^ab^ ± 0.46	2.44 ^ab^ ± 0.50	1.31 ^b^ ± 0.88
5 ppm ozone	1.75 ^bc^ ± 0.27	2.81 ^ab^ ± 0.59	3.38 ^bc^ ± 0.44	2.19 ^bc^ ± 0.53	1.13 ^b^ ± 0.58
10 ppm ozone	2.88 ^a^ ± 0.74	1.50 ^d^ ± 0.53	2.13 ^d^ ± 0.74	2.13 ^bc^ ± 0.95	1.00 ^b^ ± 0.76
25 ppm ozone	1.13 ^c^ ± 0.23	2.25 ^bc^ ± 0.53	2.75 ^cd^ ± 0.85	2.06 ^bc^ ± 0.73	1.00 ^b^ ± 0.80
10% AC	1.94 ^b^ ± 0.82	1.88 ^cd^ ± 0.44	2.38 ^d^ ± 0.52	1.75 ^bc^ ± 0.80	1.19 ^b^ ± 0.70
30% AC	2.06 ^b^ ± 0.50	2.06 ^cd^ ± 0.78	1.44 ^e^ ± 0.78	3.06 ^a^ ± 0.32	3.38 ^a^ ± 0.52
50% AC	1.13 ^c^ ± 0.79	3.00 ^a^ ± 0.93	4.13 ^a^ ± 0.52	1.50 ^c^ ± 0.65	1.44 ^b^ ± 0.56

Different letters indicate significance (*p* < 0.05) within the same column.

## Data Availability

The original contributions presented in this study are included in the article/Supplementary Materials. Further inquiries can be directed to the corresponding author.

## Supplementary Materials

The following supporting information can be downloaded at: https://www.mdpi.com/article/10.3390/foods14223820/s1, Table S1: Color values (L*a*b*) of spirulina extracts after ozone and activated carbon (AC) treatments.

## Abbreviations

The following abbreviations are used in this manuscript:

AC	Activated Carbon
AOAC	Association of Official Analytical Chemists
APC	Allophycocyanin
NIST	National Institute of Standards
PBP	Phycobiliprotein
PC	Phycocyanin
PE	Phycoerythrin
VOCs	Volatile organic compounds
PCA	Principal Component Analysis
PLS	Partial Least Squares
EFSA	European Food Safety Authority
OSHA	Occupational Safety and Health Administration
