# Supplementary material for: Deodorization of Spirulina Extracts by Ozone and Activated Carbon: Effects on Volatile Organic Compounds, Bioactive Pigments, Antioxidant Activity, and Sensory Profile
Source: Foods. 2025 Nov 7;14(22):3820. doi: 10.3390/foods14223820 (PMC12651774; doi:10.3390/foods14223820)
Supplement: Supplementary file 1 [file foods-14-03820-s001.zip › foods-3938658-supplementary.pdf]

## Supplementary data

**Table S1.** Color values (L*a*b*) of spirulina extracts after ozone and activated carbon (AC) treatments.

| Sample | L* | a* | b* |
|---|---|---|---|
| **Control** | $9.27^{g} \pm 0.14$ | $-18.92^{g} \pm 0.06$ | $15.01^{e} \pm 0.17$ |
| **5 ppm Ozone** | $44.70^{b} \pm 0.13$ | $-23.86^{f} \pm 0.08$ | $-42.87^{a} \pm 0.01$ |
| **10 ppm Ozone** | $49.18^{a} \pm 0.00$ | $-32.43^{d} \pm 0.03$ | $-35.34^{b} \pm 0.02$ |
| **25 ppm Ozone** | $28.43^{c} \pm 0.12$ | $-38.90^{b} \pm 0.07$ | $-5.99^{f} \pm 0.21$ |
| **10% AC** | $19.92^{d} \pm 0.04$ | $-40.38^{a} \pm 0.04$ | $16.64^{c} \pm 0.07$ |
| **30% AC** | $14.39^{e} \pm 0.03$ | $-34.47^{c} \pm 0.06$ | $16.18^{d} \pm 0.09$ |
| **50% AC** | $11.91^{f} \pm 0.06$ | $-28.88^{e} \pm 0.31$ | $16.38^{c} \pm 0.25$ |

Different letters indicate a significant difference ($P < 0.05$) within the same column

Color determination was performed in triplicate using Color Quest XE (Hunter Lab, Reston, VA, USA).
